# 

**Published:** 1883-10

**Authors:** 


### CONTENTS:

Art. I.—The Prone Position During Operations Upon the Jaws. By L. McLane Tiffany, M. D. ....241
Art. II.—Proceedings of the American Dental Association for 1883. ....244
Art. III.—Present Systems and the Impending Education. By A. H. Thompson, D. D. S. ....256
Art. IV.—Form and Nature of Accidents Occasioned by the Eruption of Wisdom Teeth. By Dr. Magitot. ....265
Art. V.—A Variety of Leptothrix Discovered by Dr. W. D. Miller. ....269
Art. VI.—A Few Practical Observations on the Treatment of the Pulp. By W. E. Harding, L. D. S. ....272
Art. VII.—Treatment of Pulpless Teeth. W. O. Barrett, D. D. S. ....276
Art. VIII.—Another Opinion on Celluloid. By. Dr. T. C. Howe. ....278
Art. IX.—On Certain Conditions of Dead Teeth. (Continued.) By C. S. Tomes. ....280

### EDITORIAL, ETC.

The Treatment of a Cold. ....282
The Dental Institutions of Learning. ....288

### OBITUARY.

Prof. Thomas L. Buckingham, M. D. D. D. S. ....284

### MONTHLY SUMMARY.

The Effect of Tobacco on Youth. ....287
Another Death from Chloroform. ....288

